# 

**Published:** 1884-06-20

**Authors:** 


### TABLE OF CONTENTS.

ORIGINAL AND SELECTED ARTICLES:

Menstrual Leucorrhœa Contra Indications for Vaginal Injections. by Stoyell C Parsons, M. S., M. D., 201. Diphtheria, 202. To Shorten and Lessen the Pain of Natural Labor, 203. Recent Progress in Therapeutics and Materia Medica, 207. The Health of Woman as Compared to that of Man, Especially in Relation to Marriage, 210. Method of Destroying the Fœtus in Cases of Extra-Uterine Pregnancy, 212. Hæmorrhoids—Their Treatment by Cascara Sagrada, 213. Germ Origin of Disease as Seen by a Naturalist, 216. Artificial Food For Children; Convallaria Majalis, 218.

ABSTRACTS AND GLEANINGS:

Toothache; Apomorphia in Infantile Convulsions; Sixth Cholera Report of Dr. Koch, 219. Canned Foods; Caution About Jequirity; Turpentine as a Prophylactic in Infectious Diseases, 221. Surgical Errors, 222. German Cholera Commission; Liniment for Rheumatism, 224. Boracic Acid; Rupprecht on the Treatment of Boils and Carbuncles; Quebracho in Dyspnœa, 225. Climate of Upper Georgia, 226 Enlarged Tonsils, 229. Syphilytic Test; Chloroform Albuminuria; Treatment of Fistula in Ano, 230.

SCIENTIFIC ITEMS:

The "Dugong," or Vegetarian Whale; The Balloon as a Telegraph Medium; Pasteur's Laboratory. 231. The Moon and the Weather; Photographic Progress, 232.

PRACTICAL NOTES AND FORMULÆ.

Chronic Enlargement of Spleen; Hair Tonic; An Anodyne Mixture without Opium, 233. For Dysenteric Diarrhœa; For Dysentery; For Bilious Dysentery; Asthma, 234. Prevention of Pitting in Small-pox; Scrotal Pruritus; Treatment of Pruritus Ani and Ascarides, 235.

EDITORIALS AND MISCELLANEOUS.

Association of American Medical Editors; The Yellow Fever—Reward Offered; Books and Pamphlets Received, 237. The Century Magazine; American Medical Association; The Urine in Disease, 239. Receipted; Special Notices, 240.

